# Air oxidation of sulfur mustard gas simulants using a pyrene-based metal–organic framework photocatalyst

**DOI:** 10.3762/bjnano.10.232

**Published:** 2019-12-09

**Authors:** Ghada Ayoub, Mihails Arhangelskis, Xuan Zhang, Florencia Son, Timur Islamoglu, Tomislav Friščić, Omar K Farha

**Affiliations:** 1 Department of Chemistry, McGill University, Montreal, Quebec, H3A 0B8, Canada; 2 International Institute of Nanotechnology, Department of Chemistry, Northwestern University, 2145 Sheridan Rd, Evanston, IL 60208, USA; 3 Department of Chemical & Biological Engineering, Northwestern University, 2145 Sheridan Rd, Evanston, IL 60208, USA

**Keywords:** metal-organic frameworks, oxidation, photocatalysis, singlet oxygen, sulfur mustard gas

## Abstract

We demonstrate a microporous metal–organic framework NU-400 based on a 2,7-disubstituted pyrene linker as a highly efficient photosensitizer for generating singlet oxygen and subsequent oxidative degradation of chemical warfare agents (CWAs). The high activity of NU-400 permits photocatalytic conversion of the 2-chloroethyl ethyl sulfide (CEES) mustard gas simulant into a benign sulfoxide derivative, in air, with less than 15 minutes’ half-life. This is a considerable improvement to NU-1000, based on a 1,3,6,8-tetrasubstituted pyrene unit, demonstrating how variation of the substitution pattern of a metal–organic framework linker permits modification of its photoactive behavior.

## Introduction

Sulfur mustard gas also known as mustard gas, HD, or Yperite belongs to a class of chemical warfare agents (CWAs) known as vesicants, which have detrimental effects on humans, including the blistering of skin upon contact [1]. Even at a low dosage [2], this chemical can be fatal. Although in 1993 at the Chemical Weapon Convention (CWC) 192 nations signed an agreement to outlaw the production, stockpiling, and deployment of chemical weapons, sulfur mustard gas has continuously been used against civilians and soldiers over the past several decades [3], including as recently as 2018 in Syria [4–7]. Therefore, it is imperative to design and develop novel methods for the detoxification of sulfur mustard gas in stockpiles as well as in the battle field.

**Scheme 1:**
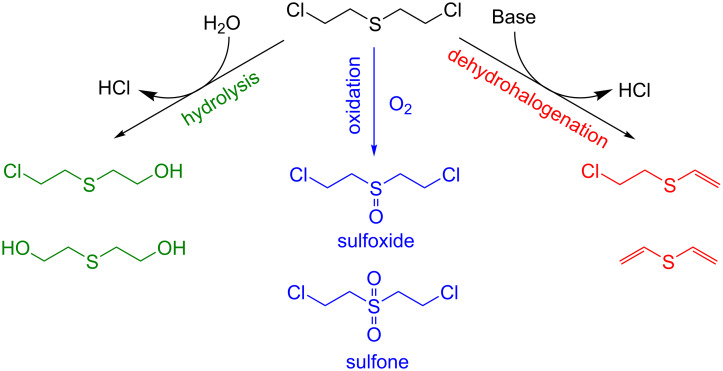
Detoxification routes of sulfur mustard gas: hydrolysis (green); oxidation to sulfoxide (blue) and dehydrohalogenation (red).

There are several routes for the detoxification of sulfur mustard gas, including: 1) hydrolysis [8–9], 2) oxidation, and 3) dehydrohalogenation [10–12] (Scheme 1). The hydrolysis route is limited to small scales because of the hydrophobicity of sulfur mustard gas. The mechanism of degradation by dehydrohalogenation mechanism is still poorly understood and not efficient enough for real-world applications. So far, the oxidative degradation [13–19] route has been shown to be the most promising but relies on the use of oxidants highly selective for the formation of sulfoxides, as further oxidation to a sulfone leads to a product of toxicity comparable to sulfur mustard gas [20].

While oxidative detoxification of sulfur mustard gas has traditionally relied on bleaching powders, such reagents come with significant drawbacks, including corrosiveness and loss of activity over time [21–22]. Ideal candidates for the oxidation of mustard gas should be mild oxidants that will selectively produce the partially oxidized sulfoxide. In that context, a promising approach is the use of singlet oxygen (^1^O_2_) generated by photosensitization of ground-state molecular oxygen (^3^O_2_) via an electronically excited chromophore. The chromophores can be compounds with significant quantum yields and whose absorption wavelengths span the ultraviolet–visible spectrum. Moreover, singlet oxygen could be generated from homogenous [23] or heterogeneous catalysts, the latter of which will allow for the ease of separation at the end of the reaction, recyclability of the catalyst, wide selection of solvent choices since solubility of the chromophore does not need to be considered, and lower probability of photobleaching [24–25].

Metal–organic frameworks (MOFs), porous crystalline materials comprised of metal nodes and organic linkers, have attracted tremendous attention in heterogeneous catalysis due to their structural and chemical tunability [26–36]. In that context, zirconium-based MOFs have demonstrated particularly high stability under a range of conditions, enabling their application for efficient, rapid hydrolytic or oxidative degradation of nerve gas agents [37–42].

Here, we describe the preparation of NU-400 [43], a zirconium-based MOF based on a judiciously chosen pyrene-based linker and utilized it as a photosensitizer for the efficient production of ^1^O_2_ and hence photocatalytic conversion of the sulfur mustard simulant 2-chloroethyl ethyl sulfide (CEES) into a benign sulfoxide product, using ambient air as the oxygen source. We selected a Zr_6_-based MOF because of its outstanding stability under a wide range of thermal and chemical conditions. As pyrene has been known as an efficient photosensitizer that is capable of producing singlet oxygen upon exposure to UV light [44–45], we anticipated that a MOF with isolated pyrene linkers would be a good candidate catalyst for the photocatalytic oxidation of sulfur mustard.

**Figure 1:**
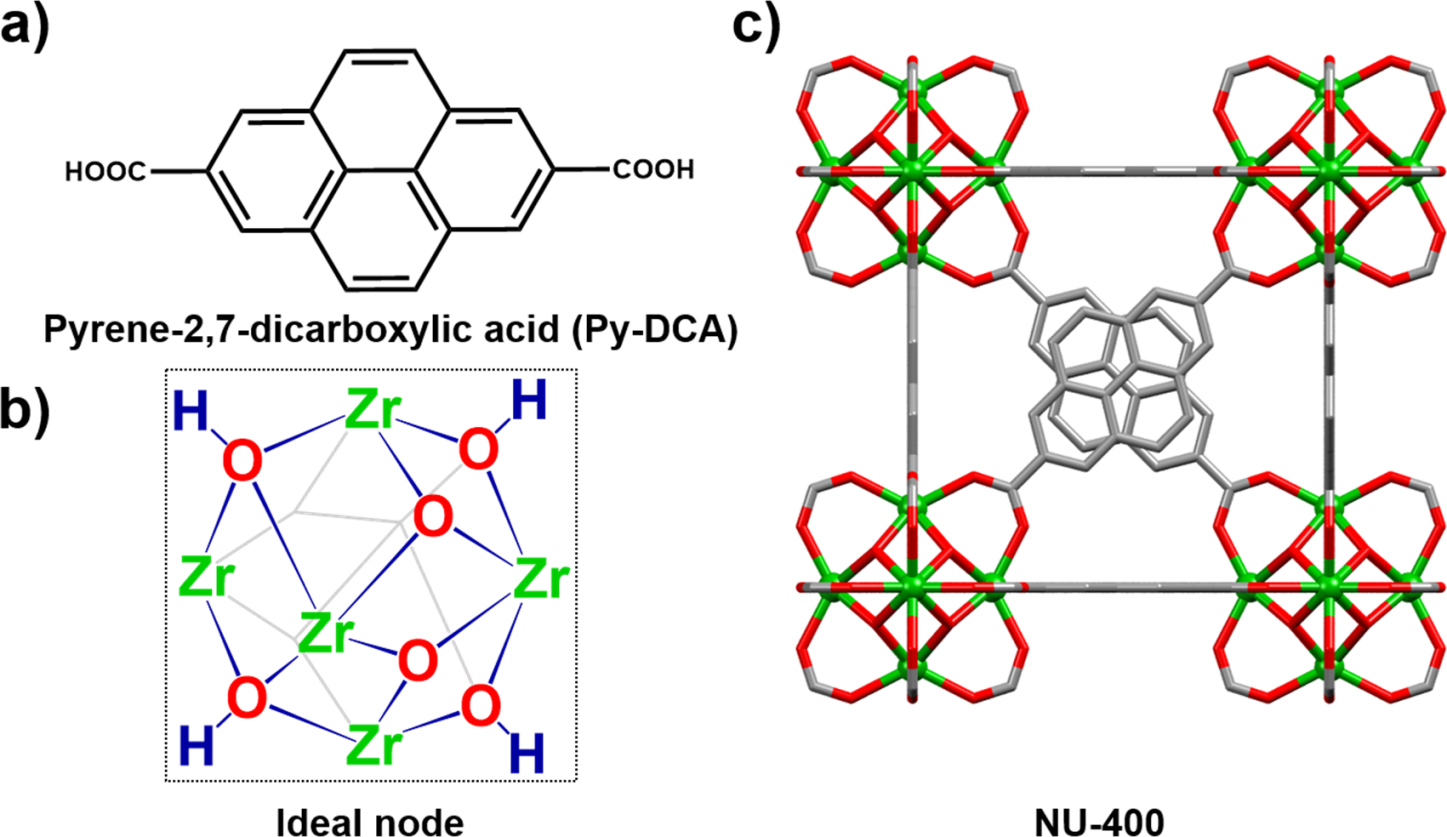
NU-400 constituents: a) the pyrene-based linker, pyrene-2,7-dicarboxylic acid and b) Zr_6_ metal node. c) Fragment of the crystal structure of NU-400, established from PXRD data. Hydrogen atoms and disorder of pyrene groups are not shown for clarity. Zr: green, O: red, and C: grey.

The NU-400 material (Figure 1) was synthesized from the pyrene-2,7-dicarboxylic acid (Py-DCA) linker, ZrCl_4_ metal salt, and acetic acid as a modulator, in DMF at 120 °C (see section S.3, Supporting Information File 1 for synthetic details). Different from the reported synthesis of pyrene-2,7-dicarboxylic acid linker [46], which required an organolithium reagent, a more benign Pd-catalyzed carbonylation reaction was utilized with 2,7-dibromopyrene as the starting material [46]. Powder X-ray diffraction (PXRD) analysis of the as-synthesized materials revealed that NU-400 is isostructural to the related UiO-67 framework based on 4,4'-biphenyldicarboxylate linkers. Subsequently, the structure of NU-400 (see section S.2.1, Table S1 in Supporting Information File 1 for crystal structure details) was established from PXRD data, by Rietveld refinement (Figure S1, Supporting Information File 1) of a model generated from UiO-67 (CCDC code WIZMAV03). The morphology of the materials was confirmed by scanning electron microscopy (SEM) images showing that bulk NU-400 material consists of octahedral crystals ranging in sizes from 1 to 5 microns (Figure S3, Supporting Information File 1). The microporous nature of NU-400 was established by N_2_ sorption measurements at 77 K, which revealed a Brunauer–Emmet–Teller (BET) surface area of 1325 m^2^/g (Figure S4, Supporting Information File 1) [44]. The pore size analysis using DFT model revealed pores of approximately 11 Å, which is suitable for diffusion of CEES molecules into the pores of NU-400.

The solid-state UV–vis spectrum of NU-400 reveals that the strongest absorption bands lie below 400 nm (see Figure S5, Supporting Information File 1), leading us to use two commercially available ultraviolet light emitting diodes (UV-LEDs) with λ_max_ = 390–400 nm as a means to excite the MOF for ^1^O_2_ production.

## Results and Discussion

**Figure 2:**
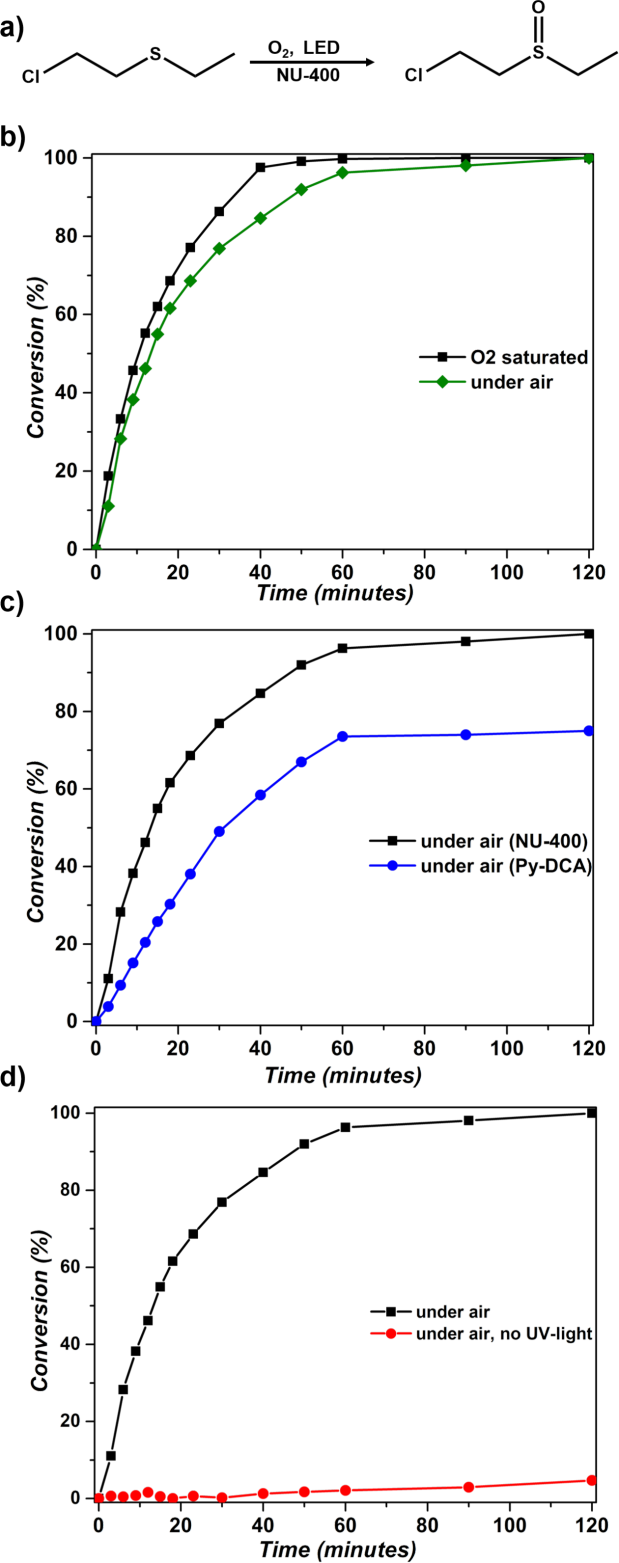
Conversion of CEES to CEESO under different conditions: (a) reaction scheme; (b) in the presence of NU-400 with O_2_ saturation and under air; (c) in the presence of Py-DCA with O_2_ saturation and under air, and (d) in the presence or absence of UV-light, under air. One mol % (based on Py-DCA) catalyst loading was used for all reactions.

As our first entry into investigating the efficiency of NU-400 as a photosensitizer for singlet oxygen generation, we used 1 mol % (2 μmol, 4.75 mg) of the MOF under oxygen saturation conditions and UV irradiation (see section S.3.3 in Supporting Information File 1 for the detailed procedure). Aliquots were taken at various time points, filtered using syringe filters and, after dilution with dichloromethane, analyzed by GC-FID to monitor the reaction kinetics. Oxidation products were analyzed by NMR spectroscopy using deuterated methanol as a solvent. Under these conditions, reaction monitoring revealed that complete and selective conversion of CEES into CEESO was achieved over a period of 50 minutes, with a half-life of 10.2 minutes (Figure 2). During control experiments designed to evaluate the significance of each parameter in the process of ^1^O_2_ production, we unexpectedly found that conducting the photocatalytic oxidation under the same conditions of irradiation, and the same MOF content, but in the absence of O_2_ saturation step, also led to complete conversion of CEES into the sulfoxide. Specifically, under such conditions the complete conversion of CEES was observed after 2 hours, with a half-life of 13.5 minutes (Figure 2). Achieving complete oxidation of CEES without the O_2_ saturation represents a milestone for the potential deployment of MOFs as an active detoxification catalyst and, consequently, we focused on detailed exploration of the activity of NU-400 in air, without oxygen purging.

Given that NU-400 is an active photocatalyst, where the pyrene-based linkers are expected to play the role of photosensitizers responsible for singlet oxygen production under UV irradiation, several control studies were performed to firmly establish the role of the linker. We explored the ability of pure linker precursor to act as the photosensitizer by performing the oxidation in the presence of 1 mol % Py-DCA (3.4 mg, 11.5 μmol) under air and in the presence of UV light, leading to a 75% conversion of CEES to CEESO after 2 hours (Figure 2). This observation implies that incorporation into the MOF structure enhanced the catalytic activity of Py-DCA, most likely due to the heterogeneous nature of the MOF, which assembles the pyrene linkers periodically within a robust three-dimensional framework, preventing their deactivation through aggregation. In all cases the ^1^O_2_ acted as a highly selective oxidant for the formation of the target sulfoxide species, as no overoxidation to form a more toxic sulfone analogue was observed (Figure S6, Supporting Information File 1). Finally, we explored the possibility of the reaction occurring in the absence of UV light. Under these conditions, in the presence of 1 mol % (12 μmol, 3.4 mg) of the MOF, no conversion of CEES was detected (Figure 2), confirming the role of NU-400 as a photocatalyst.

**Figure 3:**
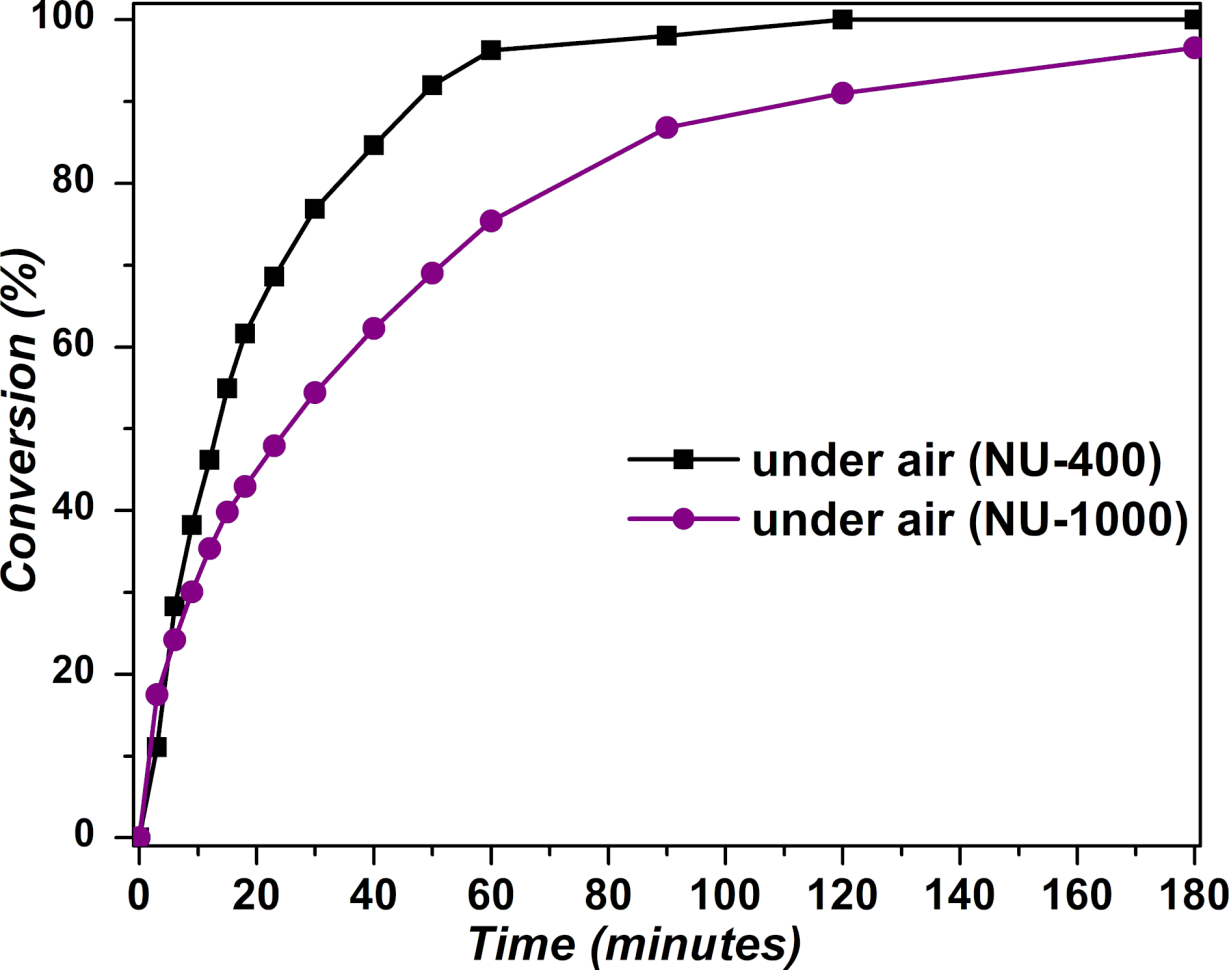
Selective oxidation of CEES to CEESO using 1 mol % catalyst of NU-400 (black) versus NU-1000 (purple).

The photocatalytic activity of NU-400 in air, without oxygen presaturation, is significantly higher compared to the previously explored mesoporous NU-1000 MOF, which is based on a different, tetratopic pyrene-based linker 4,4’,4’’,4’’’-(pyrene-1,3,6,8-tetrayl)tetrabenzoate (H_4_TBApy). Using 1 mol % (5.2 mg) NU-1000 as a photocatalyst enabled the full conversion of CEES into CEESO with a half-life of only 6.2 minutes under conditions of O_2_ saturation. However, the process was significantly slower, with a half-life of 24.5 minutes (Figure 3), when the reaction vessel was not saturated with oxygen. The superior performance of NU-400 (half-life of 13.5 minutes) under air can be attributed to the higher density of pyrene linkers in NU-400 (0.101 g/cm^3^) compared to NU-1000 (0.0506 g/cm^3^) which is responsible for ^1^O_2_ generation.

**Figure 4:**
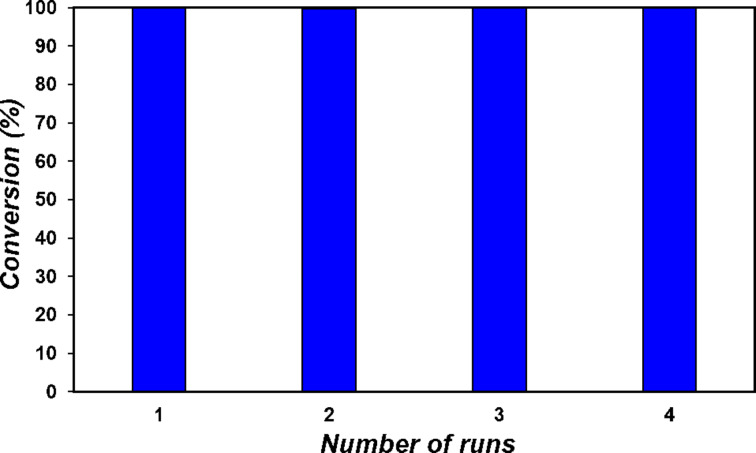
The reusability of the catalyst NU-400 MOF over four successive injections of CEES (0.2 mmol) into the same reaction vial.

Finally, we investigated to recyclability of NU-400 by adding multiple injections of CEES (0.2 μmol) into the microwave vial after one cycle of full conversion of CEES to CEESO. As the reaction was carried out using the oxygen available in atmosphere, without any additional O_2_ purging, opening the microwave vial upon the addition of CEES ensured the presence of fresh air needed for the reaction. This recyclability test was repeated three times, and the reaction progress was monitored using GC-FID in order to calculate the conversion of the reaction after each injection (Figure 4).

The photocatalytic oxidation reaction takes place without noticeable degradation of NU-400, as evidenced by PXRD analysis following four cycles of CEES to CEESO oxidation, which reveals a high degree of crystallinity (see Figure S7, Supporting Information File 1). At the same time, no overoxidation of CEES to 2-chloroethyl ethyl sulfone (CEESO_2_) was observed, as demonstrated by ^1^H NMR spectroscopy (Figure S6, Supporting Information File 1).

## Conclusion

In summary, we demonstrated NU-400, a microporous MOF based on a pyrene-2,7-dicarboxylate linker as a highly effective platform for singlet oxygen production and photocatalytic degradation of mustard gas simulant. In contrast to previously reported NU-1000, based on a 1,3,6,8-tetrasubstituted pyrene unit, which required saturation with oxygen to achieve effective high singlet oxygen production, the herein reported NU-400 is effective without oxygen saturation. The photocatalytic activity of NU-400 enabled singlet oxygen-induced conversion of CEES to CEESO with a half-life of 13.5 minutes under air, a milestone in the development of MOFs as new, highly efficient catalysts for mustard gas degradation.

## Supporting Information

File 1 Methods and materials, ligand and MOF synthesis details and additional characterization data.
