# Supplementary material for: Air oxidation of sulfur mustard gas simulants using a pyrene-based metal–organic framework photocatalyst
Source: Beilstein J Nanotechnol. 2019 Dec 9;10:2422–7. doi: 10.3762/bjnano.10.232 (PMC6941406; doi:10.3762/bjnano.10.232)
Supplement: File 1 — Methods and materials, ligand and MOF synthesis details and additional characterization data. [file Beilstein_J_Nanotechnol-10-2422-s001.pdf]

BEILSTEIN JOURNAL OF NANOTECHNOLOGY

Supporting Information

for

Air oxidation of sulfur mustard gas simulants using a pyrene-based metal–organic framework photocatalyst

Ghada Ayoub, Mihails Arhangelskis, Xuan Zhang, Florencia Son, Timur Islamoglu, Tomislav Friščić and Omar K. Farha

Beilstein J. Nanotechnol. **2019,** *10,* 2422–2427. doi:10.3762/bjnano.10.232

Methods and materials, ligand and MOF synthesis details and additional characterization data

License and Terms: This is a supporting information file under the terms of the Creative Commons Attribution License (http://creativecommons.org/licenses/by/4.0). Please note that the reuse, redistribution and reproduction in particular requires that the authors and source are credited.
The license is subject to the *Beilstein Journal of Nanotechnology* terms and conditions: (https://www.beilstein-journals.org/bjnano)

Table of Contents

**S.1**	Materials	S2
**S.2**	Characterization	S2
**S.3**	Synthesis Procedures	S4
**S.4**	Results	S6

S.1 Materials

Acetic acid (anhydrous, Sigma Aldrich), zirconium tetrachloride ($ZrCl_4$, Strem), *N,N*-dimethylformamide (DMF, Fisher), and dimethyl sulfoxide (DMSO, Fisher), Xantphos (Sigma-Aldrich), $Pd(OAc)_2$ (Strem Chemicals), N-formylsaccharin (TCI America) and KF (Sigma-Aldrich) were all purchased from commercial sources and used as received.

S.2 Instrumentation

S.2.1 Powder X-ray diffraction (PXRD)

Powder X-ray diffraction (XRD) patterns of MOFs were collected on a STOE STADI MP instrument using Kα1 Cu (λ=1.54060 Å) radiation source in transmission geometry.
The patterns were collected in the range of 4° to 48°. Analysis of PXRD patterns was conducted using Panalytical X'Pert Highscore Plus software. Experimental patterns were compared to simulated patterns calculated from single crystal structures using Mercury software package.

Crystal structure determination of NU-400 MOF was performed using TOPAS Academic V6 [1]. Crystal structure determination of NU-400 was performed by a Rietveld refinement, using the isostructural UiO-67 as starting model [2]. The peak profile was described using pseudo-Voigt function with a simple axial divergence model. The background was modelled by a 6th order Chebyshev polynomial. Coordinates of the Zr atom were refined independently, while the position and orientation of the pyrene linker was refined as a rigid body, taking into account rotational disorder of the linker. Additional oxygen atoms with fractional occupancy were introduced into the structure, and their positions refined, as a way of modelling electron density within the MOF pores, however the exact nature of the guests could not be resolved from the PXRD analysis. Crystallogrpahic parameters for the structure are summarized in Table S1, and the Rietveld fit is shown on Figure S7. Supplementary crystallographic data set for the structure is available through the Cambridge Structural Data base with deposition number 1948117. Copy of this information may be obtained free of charge, CCDC, 12 Union Road, Cambridge, CB2 1EZ, UK (fax: +44 1223 336 033; e-mail: deposit@ccdc.cam.ac.uk or http://www.ccdc.cam.ac.uk).

Table S1. Crystallographic parameters of the NU-400 structure determined by Rietveld refinement.

	**NU-400**
Formula	$Zr(C_{18}H_8O_{5.33})\bullet O_{7.81}$
M_r (g mol^{-1})	525.71
Crystal system	cubic
a / Å	26.6346(8)
b / Å	26.6346(8)
c / Å	26.6346(8)
α (°)	90
β (°)	90
γ (°)	90
V / Å^3	18894(2)
Space group	*Fm-3m*
ρ_c (g cm^{-3})	1.109
Radiation type	$CuK_{\alpha 1}$
F(000)	6266.9
R_{wp}	0.118
R_p	0.085
R_{Bragg}	0.036
χ^2	3.305

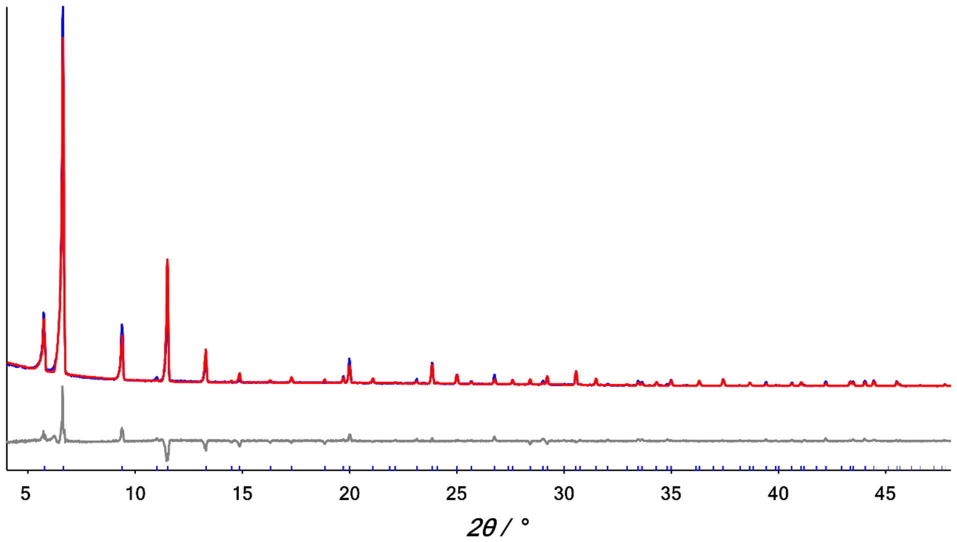

Figure S1. The final Rietveld fit for the NU-400 structure. Experimental, calculated and difference profiles are shown in blue, red and grey, respectively.

S.2.2 Scanning electron microscopy (SEM)

SEM images were collected using a Hitachi S4800-II cFEG SEM microscope the EPIC facility (NUANCE Center, Northwestern University). All samples were coated with 9 nm of OsO_4 immediately prior to imaging.

S.2.3 Nuclear magnetic resonance (NMR)

^{1}H- and ^{13}C-NMR spectra were collected on a 500 MHz (125 MHz for carbon) Bruker Avance III system equipped with DCH CryoProbe at IMSERC (Integrated Molecular Structure Education and Research Center) at Northwestern University.

S.2.4 Gas chromatography with flame ionization detector (GC-FID)

GC-FID measurements were carried out on an Agilent Technologies 7820A GC system equipped with an Agilent J&W GC HP-5 capillary column (30 m × 320 μm × 0.25 μm film thickness). All samples were filtered and diluted with dichloromethane prior to injection. Starting temperature: 70 °C, Hold: 0.5 min, Ramp: 30 °C/min, Time: 1 min, Ramp: 75 °C/min, End temperature: 300 °C. The disappearance of the reactant was calculated relative to a 0-minute time point.

S.2.5 LED irradiation

LED irradiation was performed using solderless LEDs purchased from RapidLED which were then mounted on aluminum. The LEDs were connected in series to a RapidLED 0-10V Dimmable LED driver. The irradiation setup contained two or four UV LEDs (λ_{max} = 395 nm, FWHM = 12 nm) or blue LEDs (λ_{max} = 450 nm, FWHM = 18 nm) that were mounted facing each other ~5 cm apart.

S.2.6 Activation

Samples were activated at 120 °C for 12 h under high vacuum on a Micromeritics Smart VacPrep instrument

S.2.7. *N_2 isotherm measurements*

Measurements were performed on a Micromeritics Tristar II at 77 K.

S.3. Synthesis Procedures

S.3.1 Synthesis of 2,7-pyrene dicarboxylic acid

In an argon-filled glovebox, Xantphos (0.54 mmol, 312.3 mg) and $Pd(OAc)_2$ (0.30 mmol, 67.5 mg) were added to a 150 mL heavy wall pressure vessel with a magnetic stir bar, followed by 50 mL of anhydrous N,N-dimethylformamide to dissolve the catalyst. Then 2,7-dibromopyrene [3] (7.4 mmol, 2.68 g), KF (30 mmol, 1.74 g) and N-formylsaccharin (15 mmol, 3.17 g) were added. The vessel was screw capped and heated with vigorous stirring at 80 °Cin an oil bath for three days. After cooling to room temperature, 7 mL triethylamine and 10 mL water were added, and the mixture was stirred at room temperature overnight. The solvent was removed under vacuum, then the solid was suspended in water and filtered with copious amount of water. The solid was subsequently heated and dissolved in 500 mL of 1 M NaOH aqueous solution and filtered through a cellulose membrane. The filtrate was acidified with 1 M HCl aqueous solution to about pH = 6 and the yellow precipitate was collected by filtration and washed with a large amount of water until the filtrate was neutral. The solid was rinsed with acetone and dried in vacuo to afford 1.0 g of yellow product (46% yield). ^1H NMR (NaOD and D_2O, 500 MHz) δ 8.24 (s, 4H), 7.53 (s, 4H).

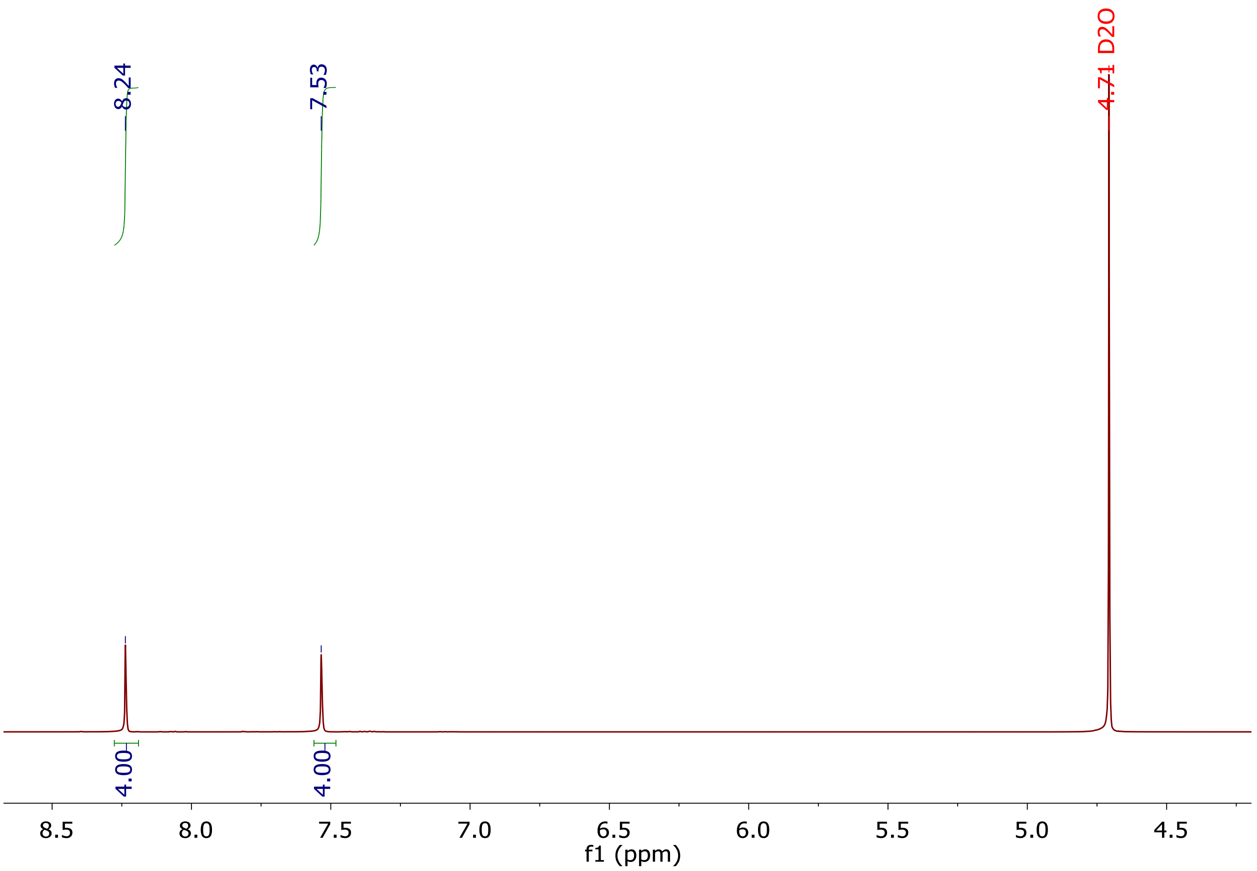

Figure S2. ^{1}H NMR spectrum of 2,7-pyrene dicarboxylic acid in NaOD and D_2O.

S.3.2 Synthesis of Defect-free NU-400

In an 8-dram vial, 2,7-pyrene dicarboxylic acid (Py-DCA, 20 mg, 0.068 mmol), triethylamine (TEA, 2 µL), DMF (16 mL), and acetic acid (6.5 mL) were added. The solution was sonicated for 20 minutes to ensure full suspension of the linker and then placed in a 100 °C oven for 15 minutes. In a separate vial, zirconium tetrachloride ($ZrCl_4$, 16 mg, 0.068 mmol) was combined with DMF (1.12 mL) and sonicated until dissolved. The $ZrCl_4$ solution was added to the linker solution and placed in a 120 °C oven for three days. The product was collected by centrifugation and washed three times with hot DMSO to remove any non-coordinated linkers. The material was washed with DMF three times followed by acetone three times. The isolated solid product was dried under vacuum for two hours at 60 °C and then activated at 120 °C under vacuum for 12 h.

S.3.3 Oxidation of CEES

For CEES oxidation experiments, the photosensitizer implemented for the reaction was dispersed in 1 mL anhydrous methanol and sealed in a 17 x 83 mm glass microwave vial. Reactions were carried either under air (without O_2 purging), or under oxygen (purging with pure O_2 for 20 min), followed by the addition of 23 µL (0.2 mmol) 2-chloroetheyl ethylsulfide (CEES) and 10 µL (0.08 mmol) internal standard (1-bromo-3,5-difluorobenzene) to the microwave vial with a 50 µL syringe. The vial was then exposed to UV LED irradiation at ambient temperature using the setup described in section ***S.2.5***. Aliquots from the microwave vial were withdrawn using a syringe at

different time points, filtered and diluted with dichloromethane. Samples were then subjected to GC-FID to monitor the reaction kinetics and final products were analyzed by NMR in CD_3OD instead of MeOH.

S.4 Results

a)

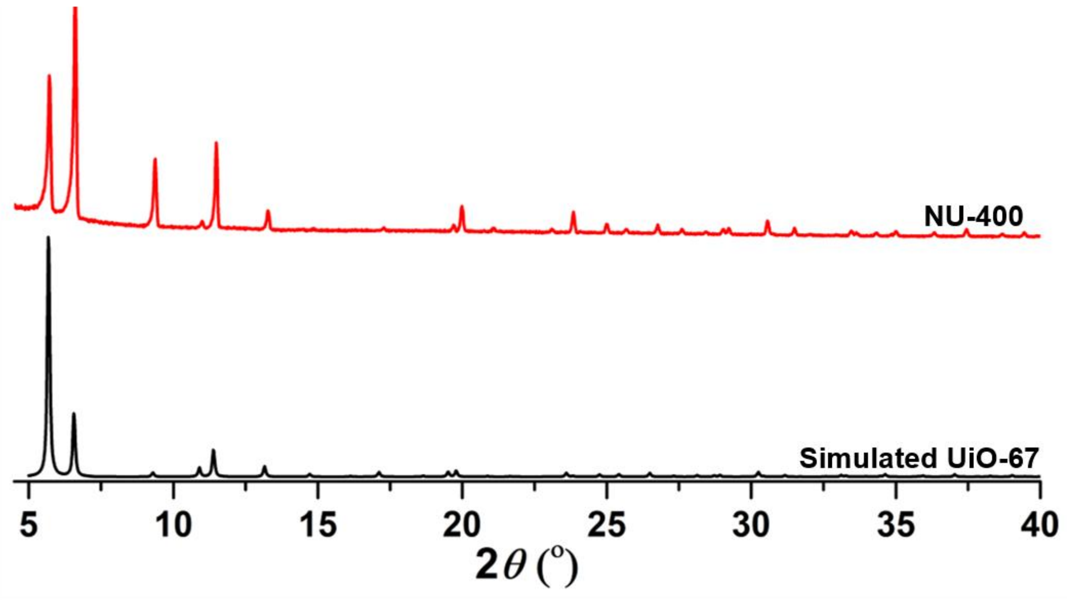

b)

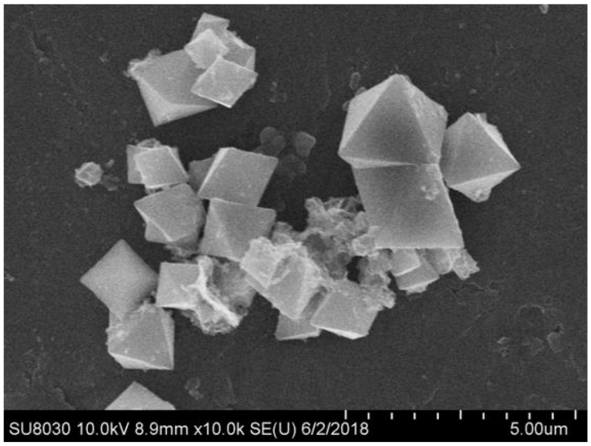

Figure S3. a) PXRD patterns of NU-400 after washing with hot DMSO with respect to the simulated powder pattern of UiO-67; b) SEM images of NU-400 after washing.

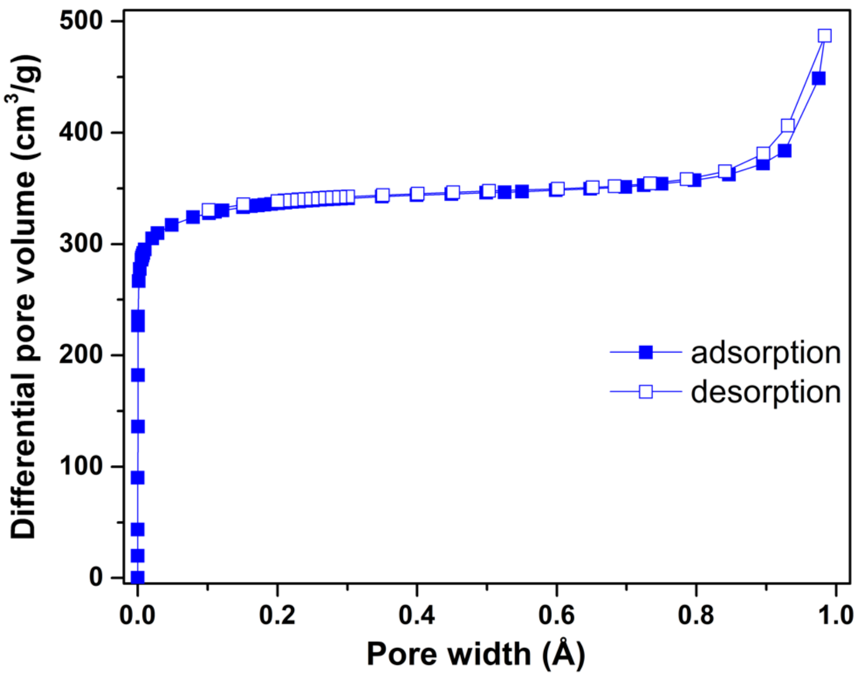

Figure S4. N_2 adsorption and desorption isotherms of NU-400 collected at 77 K.

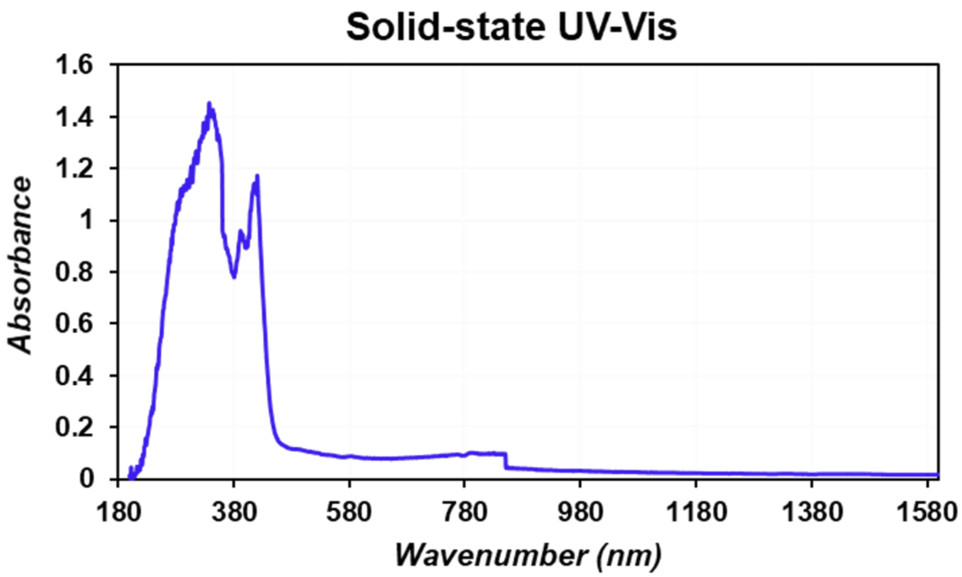

Figure S5. Solid-state UV-Vis diffuse reflectance spectrum of NU-400.

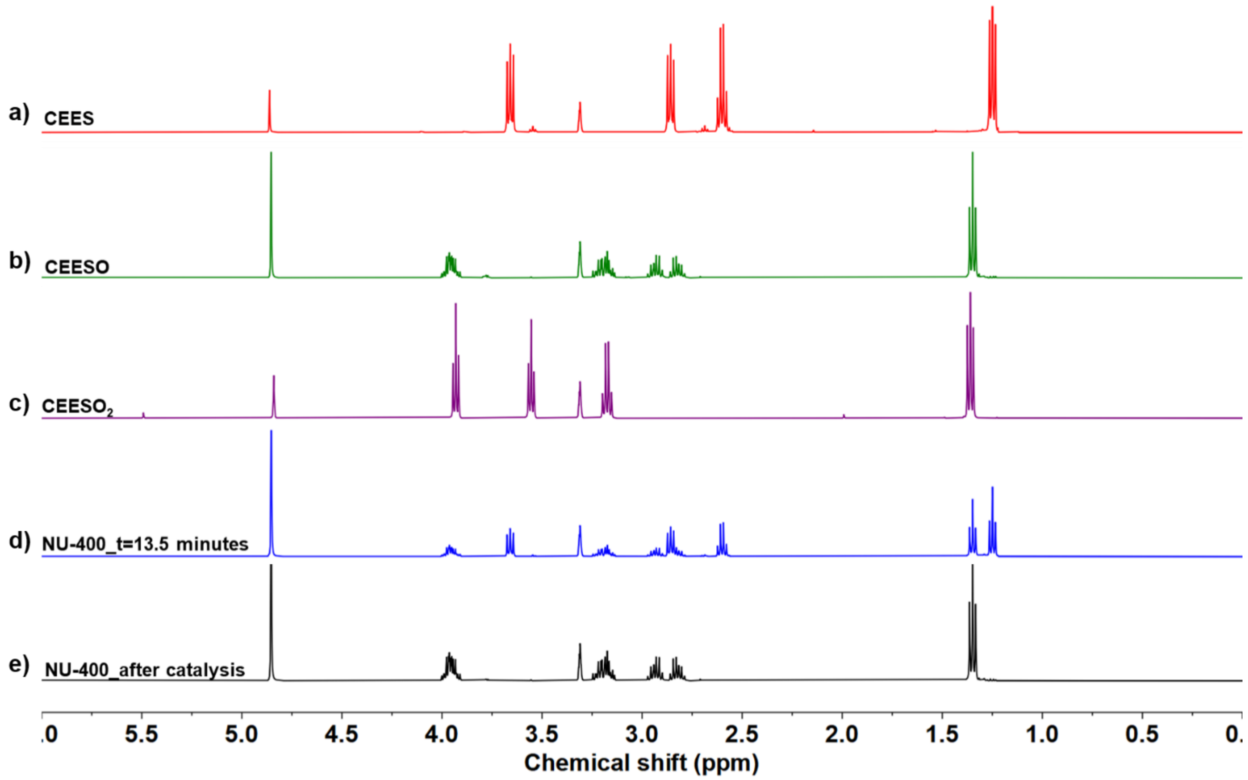

Figure S6. ^{1}H-NMR spectra of: (a) CEES, (b) CEESO, (c) $CEESO_2$, (d) CEES oxidation reaction after half of it was converted to CEESO showing a mixture of CEES and CEESO, (e) NU-400 at the end of the reaction showing CEESO only. CD_3OD used in place of methanol.

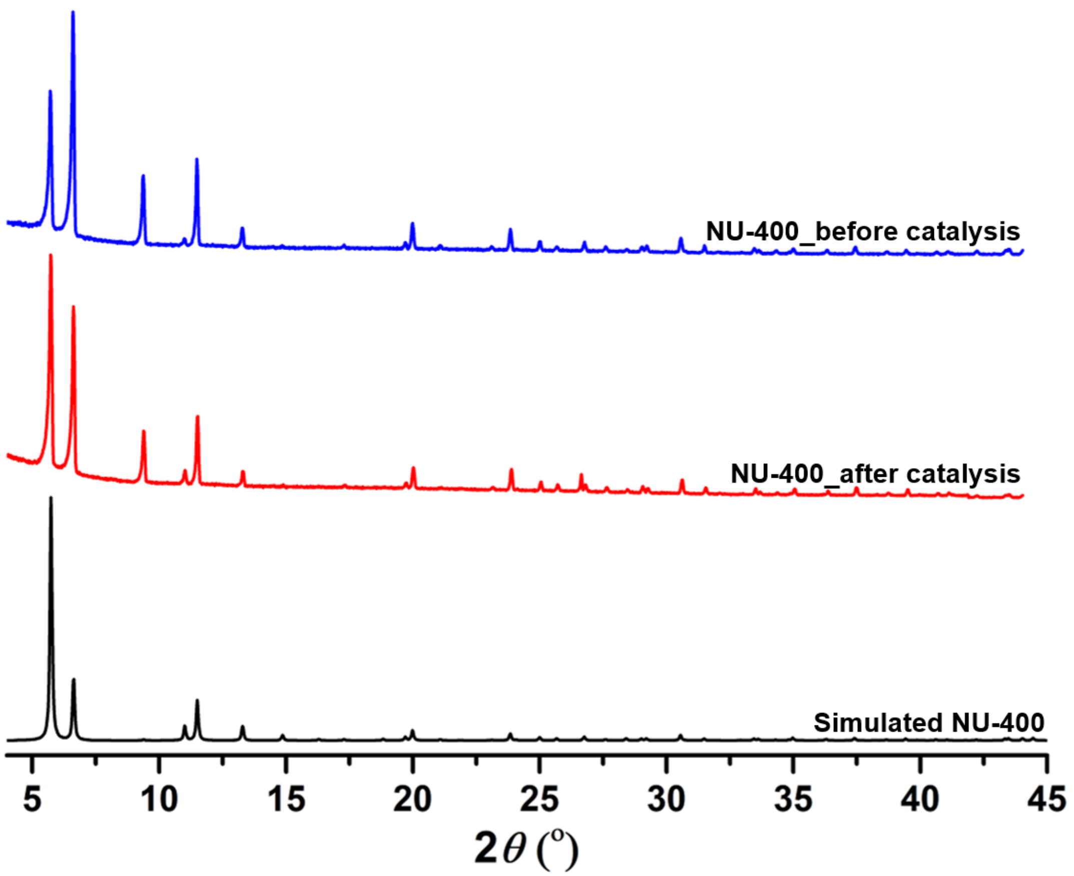

Figure S7. Powder X-ray diffraction patterns for NU-400 before and after CEES oxidation experiments in comparison to the simulated PXRD pattern of NU-400. The relative intensities of the X-ray reflections (111) and (200) at $2\theta = 5.6°$ and 6.6°, respectively, are dependent on the content of guest in the pores.

References

[1]Coelho, A. Coelho Software: Brisbane, Australia 2017.

[2] Øien, S.; Wragg, D.; Reinsch, H.; Svelle, S.; Bordiga, S.; Lamberti, C.; Lillerud, K. P. Detailed Structure Analysis of Atomic Positions and Defects in Zirconium Metal-Organic Frameworks. *Cryst. Growth Des*. **2014**, *14*, 5370−5372. dx.doi.org/10.1021/cg501386j

[3]Zhang, X.; Huang, Z.; Ferrandon, M.; Yang, D.; Robison, L.; Li, P.; Wang, T. C.; Delferro, M.; Farha, O. K. *Nat. Catal*. **2018**, *1*, 356-362. doi.org/10.1038/s41929-018-0069-6
